# Melatonin and Indole-3-Propionic Acid Reduce Surface FcγRIII/CD16-Related Parameters in Porcine Peripheral Blood Mononuclear Cells In Vitro

**DOI:** 10.3390/ijms27114898

**Published:** 2026-05-28

**Authors:** Przemysław W. Śliwka, Jan Stępniak, Małgorzata Karbownik-Lewińska

**Affiliations:** 1 Department of Endocrinology and Metabolic Diseases, Medical University of Lodz, 281/289 Rzgowska St., 93-338 Lodz, Poland; przemyslaw.sliwka@umed.lodz.pl (P.W.Ś.); jan.stepniak@umed.lodz.pl (J.S.); 2 Polish Mother’s Memorial Hospital—Research Institute, 281/289 Rzgowska St., 93-338 Lodz, Poland

**Keywords:** FcγRIII (CD16), melatonin, indole-3-propionic acid (IPA), peripheral blood mononuclear cells (PBMCs), thyroid

## Abstract

FcγRIII (CD16) is expressed by several leukocyte populations, including monocytes, macrophages, and natural killer cells, and plays an important role in IgG-mediated immune responses. Altered CD16 expression has been reported in inflammatory and autoimmune conditions, including thyroid-associated immune alterations. This preliminary in vitro study investigated whether the indole-derived compounds melatonin and indole-3-propionic acid (IPA) affect surface FcγRIII/CD16-related parameters in porcine peripheral blood mononuclear cells (PBMCs) cultured alone or with autologous thyroid follicular cells. PBMCs were left untreated or treated with melatonin or IPA, both at 50 µM, and analysed by flow cytometry at baseline and after 24 and 48 h of culture. The percentage of CD45^+^CD16^+^ cells and the CD16 mean fluorescence intensity were assessed as surface CD16-related parameters. Untreated PBMC cultures showed a time-dependent decrease in both the percentage of CD45^+^CD16^+^ cells and CD16 mean fluorescence intensity. Melatonin and IPA further enhanced this decrease compared with untreated cultures. Co-culture with thyroid follicular cells did not significantly modify CD16-related parameters under the tested conditions. These findings suggest that melatonin and IPA may modulate the surface CD16-related phenotype of porcine CD45^+^ leukocytes in vitro. The results provide preliminary evidence for the potential immunomodulatory activity of indole-derived compounds within the CD16-expressing leukocyte compartment and warrant further investigation in extended experimental models.

## 1. Introduction

Receptors of the Fcγ family are expressed on the surface of cells of the immune system where they are responsible for the binding of Fc regions of IgG-class antibodies. These receptors play a pivotal role in antibody-mediated immune responses, including antibody-dependent cellular cytotoxicity (ADCC), phagocytosis, immune complex clearance, and modulation of immune cell activation. IgG immune complexes bind to Fcγ-family molecules, triggering an intracellular signalling cascade leading to numerous downstream effects [1].

In humans, the Fcγ receptor family consists of several activating receptors (FcγRI, FcγRIIA, FcγRIIC, and FcγRIII) and one inhibitory receptor (FcγRIIB) [1,2]. Analogous receptors have been cloned and characterised in pigs [3]. Among the FcγR family receptors, FcγRIII (CD16) has garnered significant attention due to its diverse roles in immune responses. Human, mouse and pig all express CD16 on the surface of specific subpopulations of monocytes/macrophages and natural killer (NK) cells, however the distribution of this receptor is different in every species [4,5].

Altered expression of Fcγ receptors has been observed in various diseases, though the functional implications of this remain unclear [6,7]. Elevated levels of CD16 molecule expression on leukocytes have been associated with inflammatory conditions. For example, compared to healthy individuals, patients with Hashimoto’s disease exhibit significantly increased CD16 expression on peripheral blood mononuclear cells (PBMCs) [8]. A similar trend has been noted in monocytes of individuals with sarcoidosis [9], rheumatoid arthritis [10], malaria [7] and Behçet’s disease [11].

Different expressions of the CD16 molecule were also observed in immune cells infiltrating various tissues [12], including the thyroid gland. In our earlier studies we have shown, that CD16+ cells derived from thyroid focal lesions were characterised by higher surface expression of the CD16 molecule [13]. Other findings suggest that high-CD16-expressing cells are created de novo from embryonic precursors rather than being recruited from the circulatory system [12]. On the other hand, the potential upregulation of CD16 expression on cells derived from peripheral blood cannot be excluded, as transcription growth factor-β (TGF-β), present in most tissue microenvironments, is known to induce CD16 expression in monocytes [14].

It is not surprising that CD16 has been exploited as a potential therapeutic target in cancer immunotherapy and autoimmune disease modulation, particularly through monoclonal antibodies and immune-modulatory agents [15]. This may include indole-derived compounds, such as melatonin and indole-3-propionic acid (IPA), which have shown a potential role in immunomodulation; however, until today no direct impact of indole substances on CD16-mediated responses has been reported (reviewed in [16,17]).

Melatonin (N-acetyl-5-methoxytryptamine) is a widely distributed molecule found across various biological systems and synthesised by numerous living organisms. In mammals, the pineal gland serves as the principal site of melatonin production, although several peripheral organs, such as the bone marrow, lymphocytes, eyes, gastrointestinal tract, and skin, also participate in its synthesis. The production of melatonin follows a well-defined biosynthetic pathway, beginning with tryptophan as a precursor, which undergoes enzymatic modifications to form serotonin before ultimately being converted into melatonin. This process is tightly regulated by environmental light conditions, with melatonin secretion being suppressed by exposure to light and enhanced in darkness [18]. As a highly lipophilic molecule, melatonin readily traverses biological membranes, facilitating its systemic distribution and diverse physiological functions [19].

IPA shares structural similarities with melatonin, as both molecules contain a heterocyclic aromatic ring. Unlike melatonin, IPA is a deamination product of tryptophan and is primarily synthesised by the human gut microbiota. Its presence in the cerebrospinal fluid highlights its ability to cross biological barriers, originating from bacterial metabolism in the intestine. This microbial-derived compound has gained attention for its potential physiological roles, including its antioxidant properties and possible neuroprotective effects [20,21].

Both indole compounds have shown a potential role in the modulation of immunity. Melatonin, known for its immunoregulatory properties, enhances NK cell cytotoxicity, potentially through CD16-dependent mechanisms [16,22]. Similarly, IPA exhibits anti-inflammatory effects [23,24,25] that could potentially modulate CD16 signalling, reducing excessive immune activation. These suggest that indole-based compounds could serve as promising adjunct therapies in CD16-targeted immune interventions.

Interestingly, indole compounds can also directly impact various tissues, highlighting the already complex interplay between tissue and immune elements. The interaction between melatonin and the thyroid gland has been shown in previous studies, with melatonin exerting inhibitory effects on thyroid growth and hormone synthesis (reviewed in [26]). It influences thyroid function at multiple levels, including the hypothalamic–pituitary–thyroid (HPT) axis, and plays a crucial role in regulating thyroid hormone metabolism [27]. Additionally, thyroid follicular cells express melatonin receptors, while C cells are capable of synthesising melatonin under thyroid-stimulating hormone (TSH) control in rats, although the significance of this remains unclear [28]. Experimental studies have shown that melatonin administration reduces triiodothyronine (T3) and thyroxine (T4) levels in dogs and hyperthyroid rats [29], whereas hypothyroidism in rats is associated with decreased melatonin levels [30]. The protective antioxidative effects of melatonin in the thyroid have been repeatedly demonstrated in numerous studies [31], including its ability to protect against oxidative damage to membrane lipids caused by certain sodium/iodide symporter inhibitors [32]—an effect of particular importance in the thyroid, given the inherently oxidative nature of this organ [33]. These findings suggest a bidirectional regulatory mechanism between melatonin and thyroid hormone homeostasis that warrants further investigation.

In this preliminary study, we used an in vitro model to evaluate the effects of melatonin and IPA on surface FcγRIII/CD16-related parameters in porcine PBMCs. Additionally, PBMCs were cultured with or without the addition of thyroid follicular cells isolated from the same animal to evaluate the impact of basic cell-to-cell interactions and possible indirect effects of the tested indole compounds.

## 2. Results

The percentage of CD45+CD16+ cells decreased after 24 h and further after 48 h of the culture process. The initial percentage of CD45+CD16+ cells in freshly isolated PBMCs was 14.4 ± 3.81% and after 24 h and 48 h of culture, the percentage of CD45+CD16+ cells significantly decreased to 7 ± 3.22% and 2.35 ± 1.81%, respectively.

The mean fluorescence intensity of CD16 decreased after 24 h from an initial value of 3461 ± 387 to 2661 ± 433 and further decreased after 48 h, reaching a value of 2481 ± 345, while was not statistically significant compared to the value after 24 h.

When PBMCs were incubated in the presence of either melatonin or IPA, the percentage of CD45+CD16+ cells also significantly decreased after 24 h and a further decrease was observed after 48 h. However, the decrease in the percentage of CD45+CD16+ cells was significantly stronger compared to effects observed in the absence of indole substances and were as follows: 3.45 ± 1.16% and 0.25 ± 0.18%, respectively, for melatonin, and 3.55 ± 1.11% and 0.30 ± 0.28%, respectively, for IPA.

**Figure 1:**
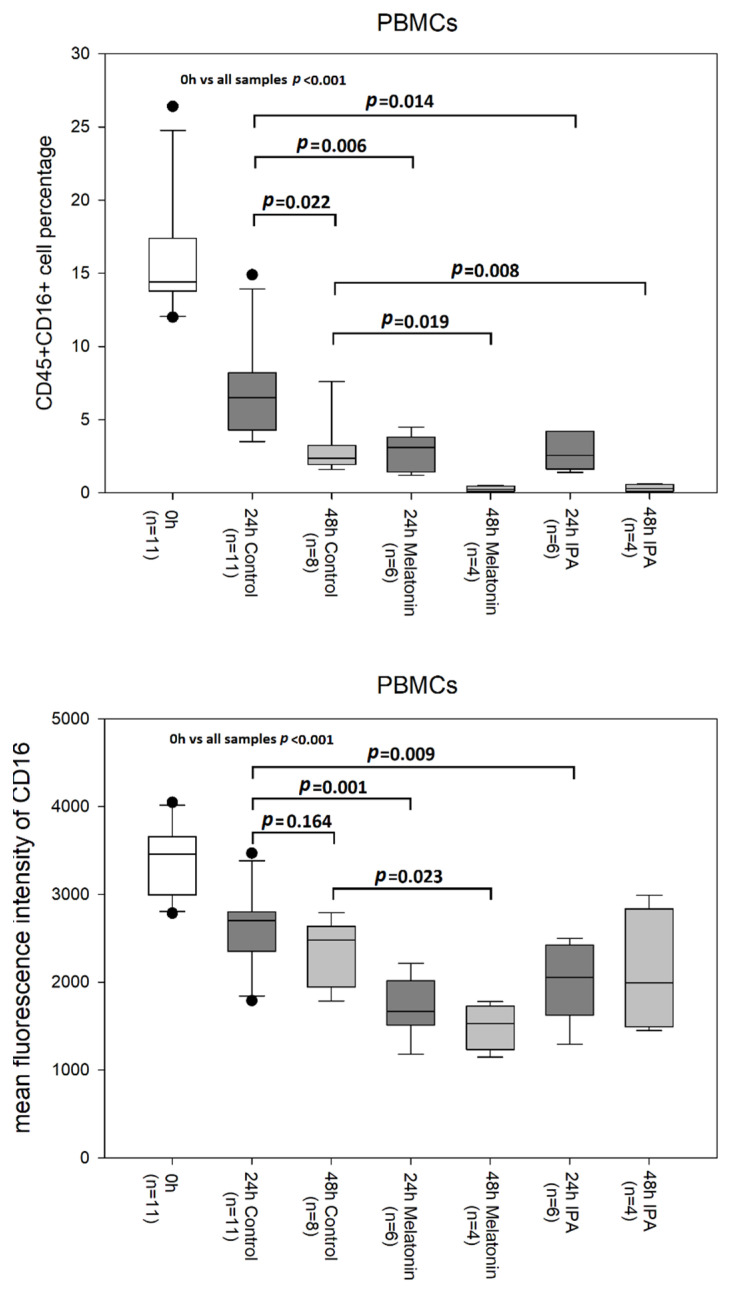
The percentage of CD45+CD16+ cells and the mean fluorescence intensity of CD16 before (0 h) and after 24 h (24 h) and 48 h (48 h) of PBMC culture. The central line of each box denotes the median and the top and bottom edges of the box show the 25% and 75% percentile, with the 5% and 95% percentiles shown by the upper and lower whiskers. PBMCs—peripheral blood mononuclear cells, IPA—indole-3-propionic acid, n—sample size, *p*—level of statistical significance.

Regarding the mean fluorescence intensity of CD16, both melatonin and IPA caused reductions similar to those observed for the percentage of CD45+CD16+ cells. The mean fluorescence intensity values decreased to 1634 ± 318 and 1534 ± 228 after melatonin treatment, and to 1878 ± 405 and 1995 ± 617 after IPA treatment, at 24 and 48 h, respectively. The decrease observed with IPA at 48 h was minor and did not reach statistical significance (Figure 1).

**Figure 2:**
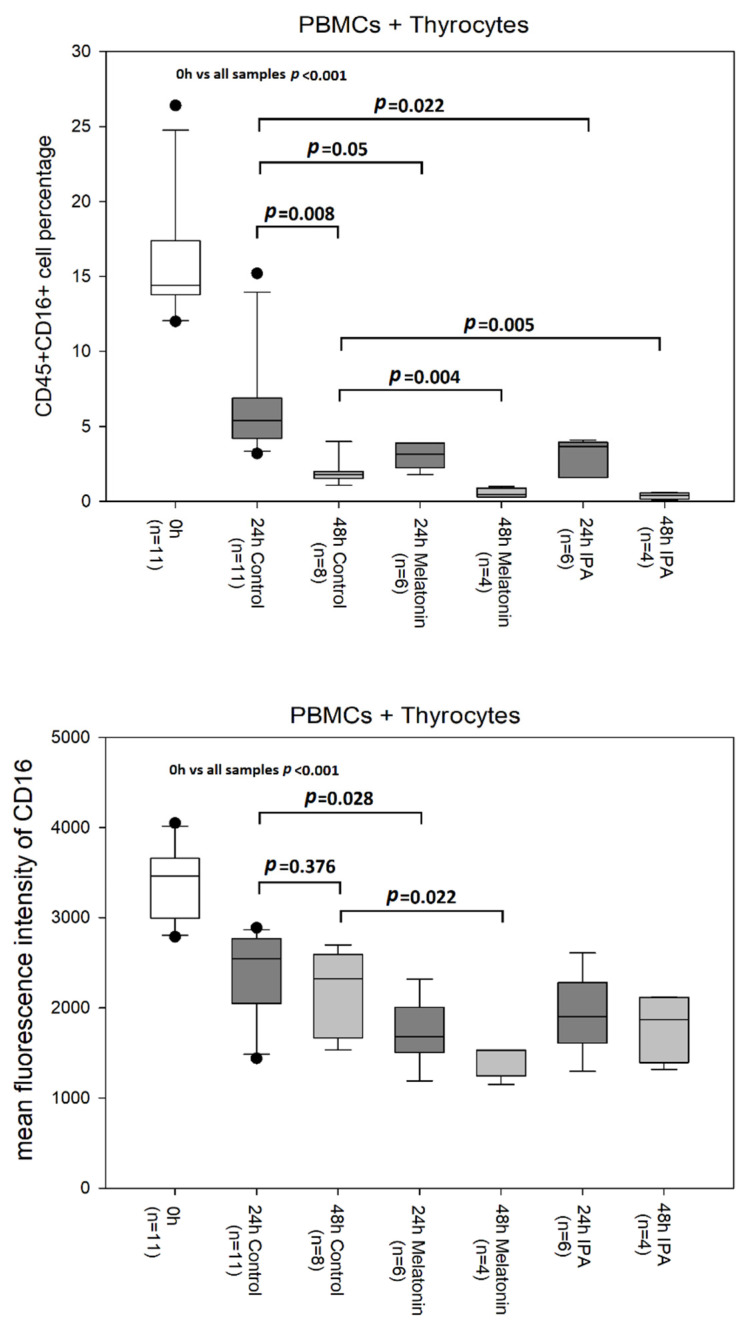
The percentage of CD45+CD16+ cells and the mean fluorescence intensity of CD16 before (0 h) and after 24 h (24 h) and 48 h (48 h) of PBMC and thyroid follicular cell (thyrocyte) co-culture. The central line of each box denotes the median and the top and bottom edges of the box show the 25% and 75% percentile, with the 10% and 90% percentiles shown by the upper and lower whiskers. PBMCs—peripheral blood mononuclear cells, IPA—indole-3-propionic acid, n—sample size, *p*—level of statistical significance.

When CD45+CD16+ cells were co-cultured with thyroid follicular cells isolated from a porcine thyroid gland the following effects were observed. After 24 h and 48 h of co-culture, the percentage of CD45+CD16+ cells significantly decreased to 5.40 ± 3.21% and 1.80 ± 0.81%, respectively, and the mean fluorescence intensity decreased to 2460 ± 452 and 2323 ± 425, respectively. Therefore, similar effects were observed regarding the cell percentage and mean fluorescence intensity after 24 h and 48 h (Figure 2).

When PBMCs were co-cultured with thyroid follicular cells in the presence of either melatonin or IPA, the percentage of CD45+CD16+ cells decreased to 3.55 ± 0.76% and 0.45 ± 0.29% for melatonin, and to 3.82 ± 1.06% and 0.40 ± 0.19% for IPA, at 24 and 48 h, respectively. This decrease was significantly stronger compared to the effects observed in the absence of indole substances. The mean fluorescence intensity values also declined, reaching 1637 ± 338 and 1529 ± 165 after melatonin treatment, and 1716 ± 415 and 1871 ± 340 after IPA treatment, at the same time points (Figure 2).

**Figure 3:**
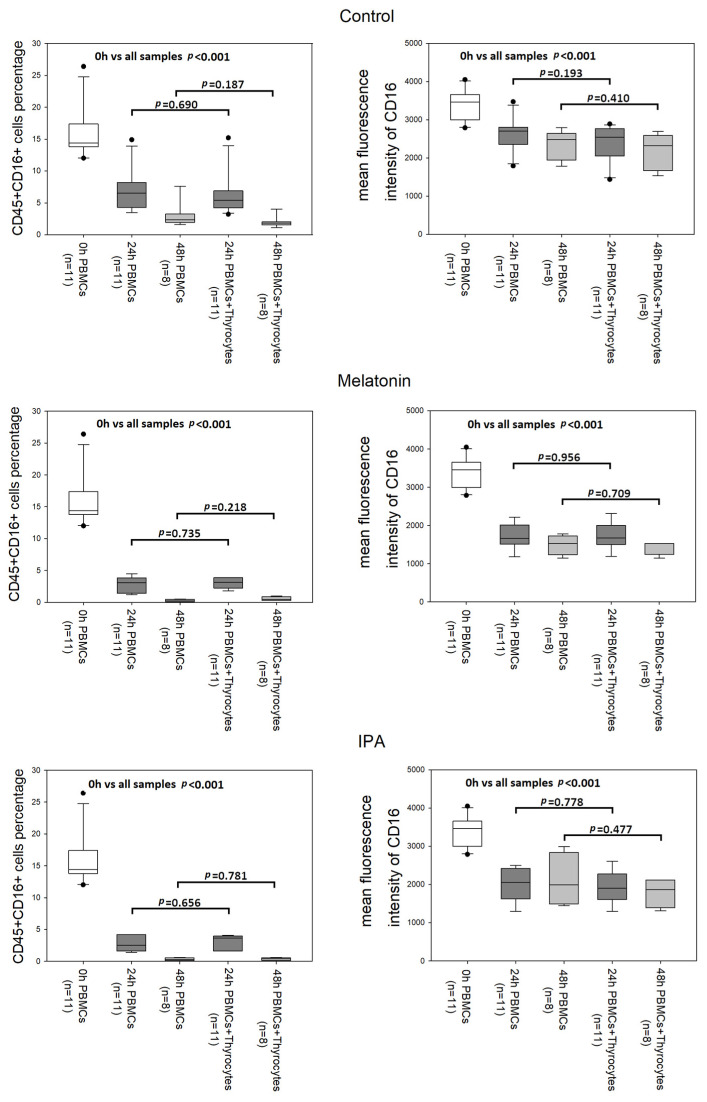
The comparison between PBMCs cultured without and in the presence of thyroid follicular cells (thyrocytes). The central line of each box denotes the median and the top and bottom edges of the box show the 25% and 75% percentile, with the 10% and 90% percentiles shown by the upper and lower whiskers. PBMCs—peripheral blood mononuclear cells, IPA—indole-3-propionic acid, n—sample size, *p*—level of statistical significance.

Of great importance is the observation that no changes were observed over time or after melatonin or IPA treatment in co-cultures with thyroid follicular cells, compared to the results obtained in the absence of thyroid follicular cells (Figure 3).

## 3. Discussion

In the present study we examined the potential changes in the characteristics of CD16 occurring with the time of the incubation and, additionally, the potential impact of indole substances, such as melatonin and IPA, on these characteristics, with additional consideration of thyroid follicular cell co-culture. The results showed a significant time-dependent decrease in both the percentage of CD45+CD16+ cells and CD16 mean fluorescence intensity, indicating a culture-associated decline in surface CD16-related parameters during ex vivo PBMC culture. Melatonin and IPA further enhanced this decline compared with untreated cultures, suggesting a possible modulatory effect on the CD16-positive leukocyte compartment under the tested in vitro conditions.

Melatonin receptors are expressed on both the membrane and nucleus of vast populations of immune cells, including T lymphocytes, B lymphocytes, monocytes/macrophages, dendritic cells (DCs) and NK cells, implicating direct regulatory effects on these immune cells [16,34]. Interestingly, the effect of melatonin on immunity is not homogeneous, but melatonin seems to act as buffering molecule. This aspect is important in the context of the cells expressing the CD16 receptor investigated in the current study, i.e., monocytes and NK cells. Melatonin has previously been shown to play a role in affecting NK cell cytotoxicity and monocyte/macrophage activation [16]. Administration of melatonin to mice in daily injections has been shown to enhance antigen presentation by splenic macrophages to T cells [35], further supporting its role in modulating immune responses. Moreover, melatonin has been reported to significantly increase IL-12 production in stimulated macrophages and in the human monocytic THP-1 cell line, although it was also shown to reduce IL-12 production in lipopolysaccharide-activated macrophages [36]. These findings highlight the dual role of melatonin in immune regulation, acting as both an enhancer and suppressor depending on the immune context.

Our findings indicate that melatonin reduced surface CD16-related parameters in PBMC cultures, which is consistent with a possible role in immune modulation under the tested in vitro conditions. One possible explanation for this effect may involve the interaction of melatonin with its receptors, potentially altering intracellular signalling cascades [19]. Another possible explanation may be related to the ability of melatonin to modulate pro-inflammatory cytokine pathways, such as interferon-γ (IFN-γ) or tumour necrosis factor-α (TNF-α) [37], which are known to sustain CD16 expression [38,39]. However, further studies aiming at characterising the molecular mechanisms of this phenomenon are needed to evaluate the effects of melatonin on immune cells, with specific focus on the differences between in vivo and in vitro mechanisms.

Additionally, our findings may also support the notion that melatonin modulates NK cell activity. The well-documented ability of melatonin to inhibit nuclear factor—kappa B (NF-κB) activation [40]; and reviewed in [16] suggests that the observed decrease in surface CD16-related parameters could be linked to altered pro-inflammatory signalling pathways. On the other hand, numerous studies have demonstrated that melatonin increases the number of NK cells in various physiological conditions. For example, exogenous melatonin administration in mice was found to elevate NK cell populations in both the bone marrow and spleen [41]. Thus, the decrease in CD16+ cells observed in our study may reflect changes within the CD16-expressing leukocyte compartment; however, without additional lineage markers, we cannot determine whether NK cells, monocytes, or other CD16-positive subsets were primarily affected.

IPA, a microbiota-derived indole compound, exhibited in the present study a similar yet slightly weaker effect on surface CD16-related parameters compared to melatonin, particularly after 48 h. The immunoregulatory properties of IPA are attributed to its anti-inflammatory action, which modulates monocyte/macrophage function and oxidative stress pathways [20,21]. While the exact mechanism underlying IPA’s influence on CD16 expression remains unclear, its reported ability to modulate pro-inflammatory responses may contribute to the observed effects.

The observed time-dependent reduction in CD16 expression indicates that the maintenance of FcγR expression requires continuous exposure to specific microenvironmental factors, such as cytokines and cell–cell interactions [42]. The substantial decline in surface CD16-related parameters observed in untreated control cultures indicates that ex vivo culture conditions themselves strongly affect the CD16-positive PBMC compartment, possibly due to the loss of physiological microenvironmental signals, including the cytokines and cell–cell interactions required to maintain FcγRIII/CD16 expression. This decrease may also reflect culture-induced phenotypic drift of primary monocytes and/or NK cells, altered activation status, receptor internalisation, proteolytic shedding from the cell surface or reduced transcription or translation of CD16. However, because the present study was based on flow-cytometric assessment of surface CD16, it does not allow us to distinguish among these mechanisms. Therefore, the observed decrease should be interpreted as modulation of the surface CD16 phenotype rather than as definitive evidence of transcriptional downregulation. Interestingly, the presence of thyroid follicular cells did not modulate, at least in our model, the decrease in CD16 expression in PBMCs. As mentioned earlier, our previous study has shown that the expression of CD16 on the surface of immune cells derived from thyroid focal lesions is higher, as compared to those derived from peripheral circulation [13]. This suggests that either the interaction between PBMCs and thyroid cells under the tested conditions was insufficient to modulate CD16 expression or that the thyroid-derived factors relevant to this process were not effectively released into the culture medium. It is also possible that PBMCs need to be stimulated by the continuous impact of circulating factors that were depleted in the in vitro settings, independent of thyroid follicular cells. It should be stressed that CD45+CD16+ cells were analysed as a broad CD16-expressing leukocyte population. Since CD45 is a pan-leukocyte marker, this gate may include monocytes, NK cells, and other CD16-positive leukocyte subsets. Without additional lineage markers, such as CD14, CD3, CD56/NK-cell markers, or granulocyte/myeloid markers, we cannot determine which subset was primarily affected by melatonin or IPA. Thus, the present results should be interpreted as changes in the overall CD45+CD16+ leukocyte compartment rather than as subset-specific effects.

The lack of a detectable effect of thyrocytes on surface CD16-related parameters suggests that, under the present experimental conditions, thyroid follicular cells did not substantially modify the response of the CD16-positive leukocyte compartment to melatonin or IPA. However, previous studies indicate that thyroid hormones and microenvironmental factors, such as TGF-β, can influence CD16 expression [14,43]. Thus, while our findings do not indicate a direct thyroid-dependent modulation of CD16 expression, potential crosstalk between thyroid and immune cells under physiological conditions cannot be excluded and requires further exploration.

From a broader perspective, our study provides preliminary evidence that melatonin and IPA may influence FcγRIII/CD16-related leukocyte phenotype in vitro. Given that altered CD16 expression has been associated with various inflammatory and autoimmune diseases, these findings could have therapeutic implications. Future studies should aim to elucidate the molecular mechanisms through which melatonin and IPA affect surface CD16-related parameters, as well as assess their effects in disease models where CD16 plays a critical role.

A limitation of the present study is the lack of a dedicated viability analysis of PBMCs across all experimental conditions and time points. Although comparable numbers of harvested/acquired cells and similar FSC/SSC profiles were observed across time points and the observed changes involved both the percentage of CD45+CD16+ cells and CD16 mean fluorescence intensity, we cannot exclude that reduced survival or selective apoptosis of CD16+ leukocytes contributed to the observed decrease.

Another limitation of the present study is the relatively small number of biological replicates in some experimental conditions. Because primary PBMC-based assays are subject to inter-individual variability, the use of n = 4 in selected comparisons limits the statistical power and generalizability of the results. Although the direction of the observed changes in the CD45+CD16+ cell percentage and CD16 mean fluorescence intensity was consistent across the analysed samples, the results should be interpreted as preliminary.

An additional limitation concerns the concentrations of melatonin and IPA used in the present study. Melatonin and IPA were applied at 50 µM, which is substantially higher than physiological circulating concentrations and should therefore be considered supraphysiological experimental concentrations. The purpose of using these concentrations was to evaluate whether melatonin or IPA are capable of modulating FcγRIII/CD16-related leukocyte phenotype under controlled in vitro conditions. Our findings should be interpreted as preliminary and require confirmation in larger independent experimental series including systematic viability and apoptosis assessment across all culture conditions and time points.

In conclusion, melatonin and IPA further enhanced the time-dependent decrease in the surface FcγRIII/CD16-related parameters observed during ex vivo culture of porcine PBMCs. Co-culture with thyroid follicular cells did not significantly modify these parameters under the tested conditions. These results suggest that indole-derived compounds may influence the CD16-positive leukocyte compartment in vitro; however, they should be interpreted as preliminary and require confirmation in larger studies including systematic viability assessment, extended leukocyte phenotyping, and mechanistic analyses.

## 4. Materials and Methods

### 4.1. Samples Collection

Porcine peripheral blood and thyroid glands were obtained from a slaughterhouse. Peripheral blood was collected into 50 mL Falcon tubes containing 110 mg of ethylenediaminetetraacetic acid (EDTA) in the first seconds of animal exsanguination done by puncture of major blood vessels at the base of the neck. The thyroid glands were collected within 20–25 min of the slaughter, placed in 50 mL Falcon tubes with Hanks’ balanced salt solution (HBSS), and kept in an ice cold container until processed. All procedures were performed/supervised by a professional veterinarian. All procedures associated with the killing of the animals were in agreement with the European Community Council Regulation (CE1099/2009).

Peripheral blood mononuclear cells (PBMCs) were isolated through gradient centrifugation at 400× *g* for 30 min using Histopaque^®^-1077 (Thermo Fisher Scientific, Waltham, MA, USA).

### 4.2. Cell Cultures

The thyroid tissue was transferred to a laminar flow cabinet, where it was carefully cleaned to remove any attached connective tissue, followed by being cut into smaller pieces, approximately 1–3 mm in size, using a sterile razor blade. After being rinsed twice with HBSS, the thyroid fragments were subjected to digestion with collagenase IV (2 mg/mL) at 37 °C for 1.5–3 h. The digested material was subsequently passed through a nylon mesh (100 μm) to separate undigested tissue remnants. Further cells were subjected to two additional HBSS washes and, therefore, centrifugation at 1000× *g* for 5 min.

Cells were cultured in a medium composed of DMEM + GlutaMAX without pyruvate (Dulbecco’s modified Eagle’s medium; Gibco), supplemented with 10% foetal bovine serum (FBS), thyroid-stimulating hormone (TSH, 1 mIU/mL), penicillin–streptomycin (100 IU/mL), and amphotericin B (2.5 μg/mL).

Cells were seeded in 6-well plates in three variants:

- Thyroid cells at a density of 1 × 10^6^ cells per mL;
- Thyroid cells at a density of 1 × 10^6^ cells per mL and PBMCs at a density of 4 × 10^5^ cells per mL;
- PBMCs at a density of 4 × 10^5^ cells per mL.

Each variant was cultured without supplementation (control) or as supplemented with melatonin (final concentration of 50 µM) or IPA (final concentration of 50 µM). These concentrations was selected as an exploratory in vitro concentrations to assess whether indole-derived compounds can influence FcγRIII/CD16-related parameters under controlled culture conditions; they was not intended to reproduce physiological circulating melatonin or IPA levels. Additionally, control samples without TSH supplementation were analysed to exclude the effect of high TSH concentrations on analysed cells.

PBMCs were analysed just before cell culture (0 h). Non-adherent cultured cells were gently harvested after 24 h (24 h) and 48 h (48 h) from the seeding time and analysed.

### 4.3. Flow Cytometry Analysis

**Figure 4:**
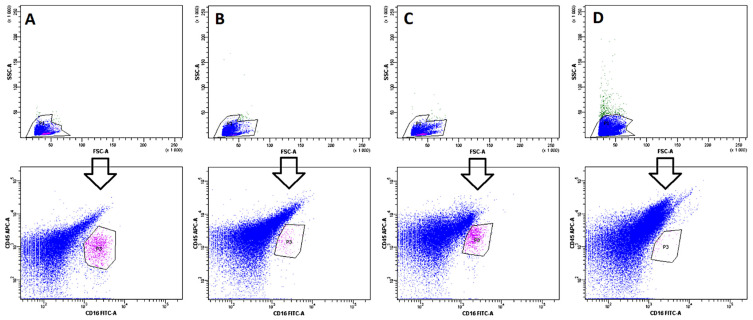
The gating strategy of flow cytometry analysis. Cells gated according to FSC (Forward Scatter) and SSC (Side Scatter) are shown on upper panels and CD45+CD16+ gating is presented on lower panels for: (**A**) PBMCs after 24 h; (**B**) PBMCs after 48 h; (**C**) PBMCs + thyrocytes after 24 h; (**D**) PBMCs + thyrocytes after 48 h.

To evaluate the changes in FcγRIII (CD16) expression on the surface of the PBMCs, cultured cells were subjected to flow cytometry. Fluorochrome-conjugated antibodies specific for porcine anti-CD45 (allophycocyanin (APC); Bio-Rad, Hercules, CA, USA) and FITC anti-CD16 (fluorescein isothiocyanate (FITC); Bio-Rad, CA, USA) were applied. Harvested cells were washed in PBS and centrifuged at 400× *g* for 5 min. Cells were then suspended in 200 µL of PBS and 2 µL of commercially prepared solution of each antibody was added followed by 20 min incubation in the dark. The appropriate isotype controls were included. To determine whether any non-specific antibody binding occurred in our experiments, we employed the Fluorescence Minus One (FMO) method and performed separate staining with isotype control antibodies. In addition, we tested the anti-human CD45 and anti-human CD16 antibodies, both of which showed no binding. We also included negative staining with thyrocytes and anti-porcine antibodies, which confirmed the specificity of our staining. Data acquisition and analysis were carried out using a BD FACSCanto II flow cytometer (Becton Dickinson, Franklin Lakes, NJ, USA). The gating strategy for flow cytometry analysis is shown on Figure 4. FcγRIII-expressing cells were identified as CD45+CD16+ and their percentage was calculated in relation to all cell singlets. The mean fluorescence intensity of the FITC channel was measured to evaluate the relative antigen abundance indicating the expression of CD16 on the surface of analysed cells.

### 4.4. Statistical Analysis

The statistical analysis and graphic representation were carried out using the SigmaPlot 15 software (Systat Software Inc., San Jose, CA, USA). The normality of the distribution was assessed using the Shapiro–Wilk test. For comparisons involving more than two groups, a one-way analysis of variance (ANOVA) was performed, followed by the Student–Neuman–Keuls’ post hoc test. For pairwise comparisons, the unpaired *t*-test was applied. In all the analyses, the results were considered statistically significant when *p* < 0.05.

## Data Availability

Data is contained within the article.
